# Wound Closure in Smoking Peripheral Arterial Disease Patients With Treatment-Refractory Ulcerations: A 12-Month Follow-up Case Series

**DOI:** 10.1177/1534734616671639

**Published:** 2016-11-15

**Authors:** Jonathan Smedley, Georgina M. Michael, Yeabsera G. Tamire

**Affiliations:** 1 Precision Podiatry, Cedar Park, TX, USA; 2 Osiris Therapeutics, Inc, Columbia, MD, USA

**Keywords:** peripheral arterial disease, cigarette smoking, treatment refractory, viable cryopreserved placental membrane

## Abstract

Despite ongoing smoking cessation efforts and optimized perfusion, failed wound closure in the presence of peripheral arterial disease (PAD) and diabetes are common. A clinical effectiveness review was conducted in actively smoking diabetic patients diagnosed with PAD, treated with serial applications of a viable intact cryopreserved human placental membrane (vCPM) (Grafix, Osiris Therapeutics Inc, Columbia, MD) for recalcitrant lower extremity ulcerations (n = 6). More than half of the patients were not candidates for revascularization. Baseline vascular status in 5 of 6 lower-extremity wounds remained unchanged throughout the entire course of vCPM treatment. Daily cigarette consumption averaged 18 cigarettes per patient. Mean wound duration and mean surface area was 53 weeks and 4.6 cm^2^, respectively. Mean number of vCPM applications and time to closure was 7.0 grafts in 7.8 weeks. There were no wound-related infections or amputations and no vCPM-related adverse events. All 6 wounds remained closed at the 12-month follow-up visit. In conclusion, vCPM demonstrated clinically effective outcomes in 6 previously nonhealing ulcerations despite ongoing smoking habits in the presence of PAD and diabetes.

As the number of nonhealing lower extremity wounds continue to rise globally, significant societal costs are seen through lost productivity and an increased financial strain on the health care system.^1-3^ In the United States alone, chronic wounds affect an estimated 2% of the population with an associated cost of care rising over $50 billion annually.^1^ Even with a multidisciplinary approach that includes advanced therapies with consistent standard of care (SOC) measures such as infection control, debridement, offloading, revascularization, compression and the promotion of patient normoglycemia, a nonhealing or treatment refractory wound may occur in as many as one-third of cases.^1-4^

The management of a treatment refractory chronic wound will exceed $9000 per year as compared with the currently estimated cost of nonrefractory chronic wound care, which ranges from $3601 to $4282 yearly per ulcer.^1,5^ Common conditions such as peripheral arterial disease (PAD), chronic venous insufficiency (CVI), diabetes, and inhaled tobacco dependence, have been linked with the development of treatment refractory wounds.^6-8^ Together, these comorbidities lead to pathophysiologic abnormalities that further complicate ulcer management and pose a challenge to the wound care specialist worldwide.^2,3^

In particular, PAD, diabetes, and active smoking alongside the presence of a nonhealing lower extremity wound represent the greatest risk for ongoing tissue loss, infection, amputation, and potential mortality.^6,9-11^ Smoking, a well-known deterrent to cutaneous healing, is often a part of a patient’s medical history.^12-18^ The presence of PAD and diabetes together with smoking lead to vasoconstriction cumulative with each cigarette that is smoked, and to oxygen and nutrient deficiencies in tissues that independently contribute to the formation and exacerbation of nonhealing ulcerations.^19-23^

Revascularization is considered the gold-standard for promoting wound closure in patients with PAD.^2^ Despite ongoing smoking cessation efforts and optimized perfusion, failures of wound closure in the presence of PAD and diabetes are common. Rates of complete lower extremity wound closure may be as low as 45% following procedures such as open surgical bypass or endovascular intervention.^20^ Delayed healing and ongoing tissue loss in the affected limb may continue to occur, even with technically successful revascularization surgeries.^4,20,24^ Forty percent of initially healed patients will also experience ulcer recurrence within the first year.^4^ Nearly three-fourths of such wounds result in major amputation due to inadequate healing.^4,20^ Even with the available advanced wound care products, technologies and improved methods of accurate diagnosis, prolonged duration of treatment and an increased morbidity related to infection and amputation continue to occur.^2,3,25,26^

## Materials and Methods

### Study Design and Population

The purpose of this study is to present the clinical outcomes associated with the use of Grafix (Osiris Therapeutics, Inc, Columbia, MD), a viable intact cryopreserved human placental membrane (vCPM), for the outpatient management of refractory lower extremity wounds in actively smoking PAD patients. A single center retrospective chart review was conducted on all patients with chronic wounds managed with vCPM during a 1-year period (July 2014 to July 2015). Because of the retrospective nature of data collection, an internal review board approval was not required for this case series analysis.

Individual patient consents were obtained for the use of all de-identified materials. Selection for subject inclusion in this analysis was based on the following criteria: (1) presence of a nonhealing lower extremity wound with a known diagnosis of moderate to severe PAD, (2) a vascular status assessment to confirm the diagnosis of PAD and potential for revascularization, (3) an ongoing cigarette smoking habit, and (4) previous SOC treatment failure in addition to advanced therapies.

From the population of vCPM-treated patients (N = 86), a subset of 4 males and 1 female (n = 5) with 6 wounds met the prespecified inclusion criteria. In addition to ankle brachial index (ABI) values, the Fontaine classification system was used to classify the severity of PAD.^27-30^ Clinical manifestations of PAD ranged from moderate to severe intermittent claudication in conjunction with minor tissue loss and nonhealing ulcerations. The classification of a wound as *recalcitrant* depended on a minimum previous treatment duration of ≥120 days (4 months) characterized by a prior failure to respond SOC and ≥1 advanced therapy regimens.^6^

### Patient Evaluation and Wound Management

Prior to the initiation of vCPM treatment, all patients were evaluated for the potential to improve their lower extremity perfusion through endovascular or open surgical intervention. All patients presented with claudication as a clinical symptom. Three subjects were categorized ineligible for revascularization, the remaining 2 patients were scheduled for invasive procedures: One patient received percutaneous stenting subsequent to treatment with vCPM; and the second patient underwent surgical revision of a previous femoral-arterial bypass graft, occurring day 30 after initiation of vCPM treatment.

All patients were managed with a multidisciplinary approach from podiatry, nursing, vascular surgery, nutrition, and endocrinology. Smoking cessation treatment was offered during every visit, including pharmacotherapy and nicotine replacement therapy.^31^ Appropriate dietary and blood glucose control was promoted and monitored in all diabetic patients.^32^ Wounds received weekly SOC treatment, including selective wound debridement, offloading, infection control, and exudate management.^33^ Modified compression dressings were applied for all lower extremity wounds of mixed arterial-venous etiology.^34^

### Clinical Effectiveness Analysis

Evaluation of clinical effectiveness in vCPM-treated patients included (1) the incidence of complete wound closure, (2) the individual and mean percentage area reduction (PAR) in wound surface area at 4 weeks of ≥50%, (3) the mean time to closure, (4) the mean number of grafts required for closure, and (5) adverse events, defined as any wound-related amputation or infection occurring during the vCPM treatment phase. Patients were also assessed at a 12-month follow-up in order to evaluate all vCPM-related wound closures.

## Results

The patient demographics and baseline wound characteristics are summarized in Table 1. Per Fontaine classification for PAD, 4 patients (80%) were categorized as stage IIa/b while the remaining patient was categorized as stage III. Daily cigarette smoking habits ranged from 0.5 to >1 pack per day (PPD) with a mean consumption of 18 cigarettes per day. Baseline surface area of the wounds (n = 6) ranged from 1.0 to 9.7 cm^2^ with a mean of 4.6 cm^2^ (SD 3.4). Average wound duration was 53 weeks (370.7 days; SD 346.2; median 220; range 121-1114 days). After initiation of treatment with vCPM, closure occurred in a mean time of 7.8 weeks (SD 4.4; range 2-15.4 weeks) with 7.0 applications (SD 3.8; range 2-14).

**Table 1.** Baseline Patient Demographics, Wound Characteristics, and Mean Study Outcomes.

Patient (Sex)	Age (Years)	BMI (kg/m^2^)	ABI	Fontaine Classification	Revascularization Status	Cigarettes (PPD)	Study Wound No.	Wound Size (cm^2^)	Wound Duration (Days)	Wound Etiology	Wound Location	4-Week PAR (%)	vCPM Applications	Time to Closure (Weeks)
1^a^ (F)	56	32.1	0.6	Stage IIb	Right superior femoral artery stent after wound closure	1	1	1.0	183	Ischemic diabetic foot ulcer	Right fifth digit	N/A	2	2
2^a^ (M)	63	39.5	0.5	Stage III	Left femoral-popliteal bypass revision after 4 vCPM applications	1	2	8.8	121	Diabetic with mixed venous and arterial disease	Left anterolateral leg	76.24	5	6
3 (M)	43	28.5	1.0	Stage IIa	Not a candidate for revascularization	1	3	9.7	256	Mixed venous and arterial disease	Right anterolateral leg	24.5	9	11
			0.8				4	3.2	412	Mixed venous and arterial disease	Left dorsal midfoot	98.75	5	5
4^a^ (M)	64	25.1	0.7	Stage IIb	Not a candidate for revascularization	1+	5	3.0	1114	Neuroischemic diabetic foot ulcer	Right plantar forefoot	63.7	14	15.4
5^a^ (M)	66	29.4	0.5	Stage IIa	Not a candidate for revascularization	0.5	6	1.9	138	Ischemic diabetic foot ulcer	Right dorsal forefoot	83.87	7	7.14
**Mean**	**58.4**	**30.92**	**0.7**			**0.9**		**4.6**	**370.7**			**69.4**	**7**	**7.8**

Abbreviations: ABI, ankle brachial index; BMI, body mass index; F, female; M, male; N/A, no applicable; PAR, percentage area reduction; PPD, packs per day (1 PPD = 20 cigarettes); vCPM, viable intact cryopreserved human placental membrane.

a Diabetic.

Five of the ulcers met the minimum 28-day treatment time for calculation of the mean 4-week PAR. Four of the ulcers surpassed the minimum benchmark for clinically effective treatment progress (≥50%), resulting in mean PAR of 69.4% (SD 25.2%; range 24.5%-98.8%). Study wound 1 received only 2 serial graft applications (day 0 and day 7), and reached a 75% PAR at the first follow-up visit and complete wound closure by day 15. The patient with study wound 3 did not return for a period of 3 weeks after day 14 of initial treatment with vCPM (applications: day 0, day 7, and day 14). Study wound 3 had a 2-week PAR of 25.6%. This 20-day disruption of care resulted in an increased wound surface area. Wound size reduction was observed when vCPM applications were resumed, resulting in a 50.4 % PAR by day 49. Adjusting for the 20-day treatment gap, study wound 3 demonstrated a ≥50% in wound size with 4 active weeks of treatment with vCPM. This adjusted PAR was not included in the mean % PAR calculations.

## Selected Case

### Patient 3

A 43-year old male patient (body mass index 28.5 kg/m^2^) presented with 2 separate wounds on the left and right lower extremities, etiology included mixed venous and arterial disease (Patient 3, see Table 1). vCPM applications were initiated after both wounds had been present for 256 days. The patient had bilateral nonpalpable dorsalis pedis and posterior tibial pulses. ABI ranged from 0.8 on the right lower extremity to 1.0 on the left lower extremity, Fontaine classification IIa. The patient was not considered a candidate for revascularization procedures due to noncompliance, procedure refusal, and ongoing intravenous drug abuse. Past medical history included moderate PAD, hypertension, dyslipidemia, substance abuse, and deep vein thrombosis. Cigarette smoking habit equaled 1 PPD. Past treatment failures included acellular and cellular skin substitutes.

**Figure 1.**
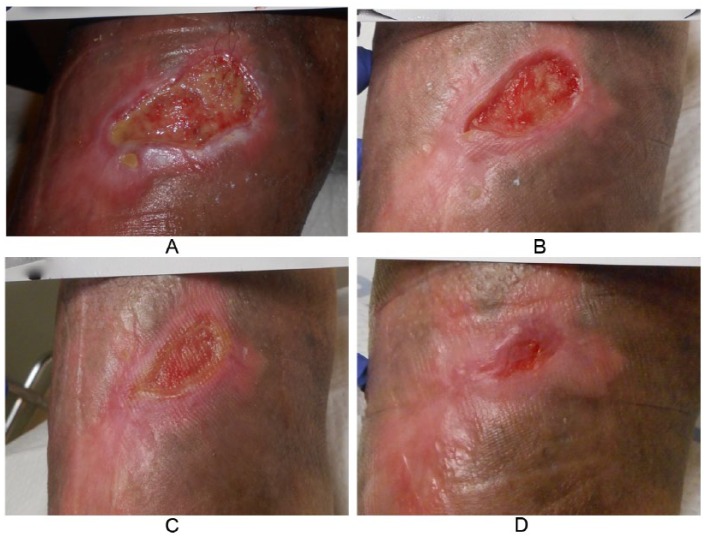
Study wound 3. (A) Baseline: 9.7 cm^2^ wound of the right anterolateral tibia with a duration of 256 days prior to application of vCPM. (B) Day 49: 50.5% PAR after 5 applications (C) Day 56: 71.5% PAR after 6 applications. (D) Day 77: Final wound closure following 9 vCPM applications. PAR, percentage area reduction; vCPM, viable intact cryopreserved human placental membrane.

**Figure 2.**
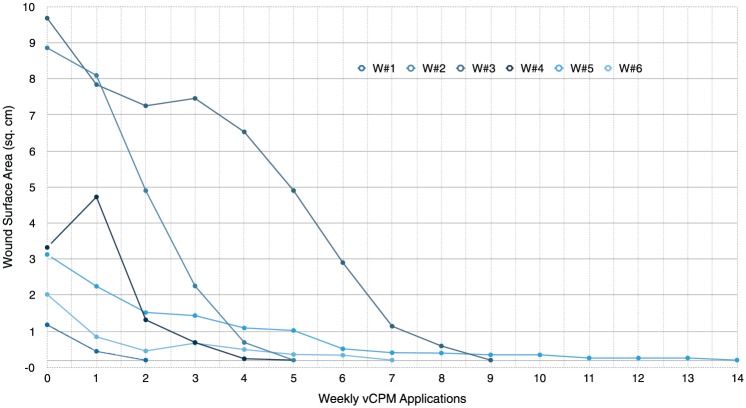
Progressive reductions in wound surface area with serial viable intact cryopreserved human placental membrane (vCPM) applications.

- Study wound 3: 9.7 cm^2^ wound of the right anterolateral tibia with a previous duration of 256 days. Nine serial applications of vCPM were done with complete closure at day 77 (Figures 1A-D and 2).
- Study wound 4: 3.2 cm^2^ wound of the left dorsal foot with a previous duration of 412 days. Complete closure at day 36 was achieved after 5 serial applications of vCPM.

## Discussion

The clinical effectiveness of an advanced therapy can be assessed by the incidence and time to complete wound closure. The management-related clinical progress can also be monitored through reductions in wound size with treatment. The objective of this study was to evaluate the clinical effectiveness of vCPM in subjects with both pathophysiological and patient behavior–related challenges that prevent wound closure, even after reperfusion optimization.

Diabetes and an active smoking habit are associated with the most significant increases in treatment time and financial expenditures related to wound management.^1,35^ All patients included in this review were long-term heavy smokers with a mean consumption of nearly 1 PPD. Four patients with 5 wounds were also diabetic. These high-risk patients are typically excluded from randomized controlled clinical trials. Nonclosed wounds in such patients represent a prominent risk factor for infection leading to hospitalization and lower limb amputation.^4,9^

Although most individuals are initially considered for revascularization procedures, factors such as age, patient consent, comorbidities, and the extent and pattern of vessel occlusion may limit surgical reperfusion options.^9,36^ Many clinicians are left to pursue outpatient management instead of the more aggressive surgically based wound closure strategies.^4,9,24^ More than half of the patients included in this study were not candidates for revascularization procedures. As a result, baseline vascular status and ABI values for 5 of 6 lower extremity wounds remained unchanged throughout the entire course of vCPM treatment. Although the patient with study wound 2 underwent a surgical revision of his femoral-popliteal bypass on treatment day 30, a 76.2% reduction in wound surface area was recorded during the initial 4 weeks of vCPM treatment.

Even in wounds with a mixed etiology such as diabetic with arterial and venous insufficiency, literature indicates that surrogate markers with predictive value for clinical effectiveness, and thus for healing, can be identified.^37^ Lavery et al^38^ reported on the predictive value of early wound progression, where a ≥15% PAR at week 1 could be used to identify the likelihood of healing by 16 weeks versus the need for considering a change in treatment. In this study, an early shift from recalcitrance to a mean 15.8% PAR at week 1 of vCPM applications is consistent with predicting positive clinical outcomes. The continued mean reductions in surface area were 55.3%, 65.2%, and 79.3%, at weeks 2, 3, and 4, respectively. This progressive trajectory toward 100% closure is also considered indicative of long-term healing potential.^32,38-41^

A reevaluation of treatment regimen is also recommended for wounds with PAR <50% by week 4.^39-42^ Eighty-three percent (5/6) of the wounds were treated up to day 28 for calculations of surface area reduction with a mean 28-day PAR of 69.4%. Eighty percent of these wounds demonstrated a correlation between the ≥50% at the 4-week point and subsequent closure. Treated for a total of 11 weeks, study wound 3 may be categorized as an outlier since failure to meet the minimum benchmark for surface area reduction was due to a 20-day gap between vCPM applications 3 and 4, thus preventing documentation of wound size and/or surface area during this period of time.

Infections substantially increase the morbidity and mortality associated with open wounds, particularly diabetic foot ulcerations and pressure ulcers.^30^ PAD patients are almost 90 times more likely to receive lower extremity amputations once infection is present.^4^ PAD severity is independently correlated with reductions in primary healing while simultaneously increasing the rates of amputation and mortality.^10,41^ Despite a mean ABI of 0.7 with stage II/III PAD (per Fontaine classification), there were no treatment-related infections or wound-related amputations reported during this study. No vCPM-related adverse events were reported. In order to assess the quality of wound closure, patients were followed for 12 months. No subjects were lost to follow-up. All 6 wounds in 5 patients remained closed at the 1-year follow-up evaluation. Thus, we report durable wound closure versus the recurrence typically associated with transient wound coverage.^32^

In general, biological dressings or wound covers have not been shown to be vastly successful.^2^ However, vCPM represents an emerging tissue preservation technology that should be explored for its potential benefits in the management of nonhealing wounds.^2^ A prospective multi-center randomized clinical trial found vCPM to be beneficial for diabetic foot ulceration (62% complete wound closure versus 21% with SOC alone).^33^ While clear limitations such as the lack of smoking controls do exist in the design of this retrospective case series, the outcomes in this study suggest that vCPM should be considered in the wound management of PAD patients with otherwise limited options for reperfusion. vCPM may contribute to wound closure in high-risk smoking patients with a history of SOC and advanced treatment failures. vCPM and other innovative technologies aimed at improving patient outcomes should be continuously examined through multiple levels of evidence, supported by both clinician and researcher.
